# PRV-1 Infected Macrophages in Melanized Focal Changes in White Muscle of Atlantic Salmon (*Salmo salar*) Correlates With a Pro-Inflammatory Environment

**DOI:** 10.3389/fimmu.2021.664624

**Published:** 2021-04-29

**Authors:** Muhammad Salman Malik, Håvard Bjørgen, Ingvild Berg Nyman, Øystein Wessel, Erling Olaf Koppang, Maria K. Dahle, Espen Rimstad

**Affiliations:** ^1^ Section of Virology, Faculty of Veterinary Medicine, Norwegian University of Life Sciences, Ås, Norway; ^2^ Section of Anatomy, Faculty of Veterinary Medicine, Norwegian University of Life Sciences, Ås, Norway; ^3^ Department of Fish Health, Norwegian Veterinary Institute, Oslo, Norway

**Keywords:** Atlantic salmon, black spots, macrophage polarization, *Piscine orthoreovirus*, red spots

## Abstract

Melanized focal changes in white skeletal muscle of farmed Atlantic salmon, “black spots”, is a quality problem affecting on average 20% of slaughtered fish. The spots appear initially as “red spots” characterized by hemorrhages and acute inflammation and progress into black spots characterized by chronic inflammation and abundant pigmented cells. *Piscine orthoreovirus* 1 (PRV-1) was previously found to be associated with macrophages and melano-macrophages in red and black spots. Here we have addressed the inflammatory microenvironment of red and black spots by studying the polarization status of the macrophages and cell mediated immune responses in spots, in both PRV-1 infected and non-infected fish. Samples that had been collected at regular intervals through the seawater production phase in a commercial farm were analyzed by multiplex fluorescent *in situ* hybridization (FISH) and RT-qPCR methods. Detection of abundant inducible nitric oxide synthase (iNOS2) expressing M1-polarized macrophages in red spots demonstrated a pro-inflammatory microenvironment. There was an almost perfect co-localization with the iNOS2 expression and PRV-1 infection. Black spots, on the other side, had few iNOS2 expressing cells, but a relatively high number of arginase-2 expressing anti-inflammatory M2-polarized macrophages containing melanin. The numerous M2-polarized melano-macrophages in black spots indicate an ongoing healing phase. Co-localization of PRV-1 and cells expressing CD8^+^ and MHC-I suggests a targeted immune response taking place in the spots. Altogether, this study indicates that PRV-1 induces a pro-inflammatory environment that is important for the pathogenesis of the spots. We do not have indication that infection of PRV-1 is the initial causative agent of this condition.

## Introduction

Melanized focal changes in the white skeletal muscle of farmed Atlantic salmon (*Salmo salar*), “black spots”, has emerged as a phenomenon that is found on average in 20% of the Atlantic salmon slaughtered at Norwegian processing plants (1). Fish affected with spots appear clinically healthy, and the condition is therefore regarded as a quality problem rather than associated with a disease state. Most melanized changes locate to the cranio‐ventral and mid‐ventral parts of the fillet, which could indicate an anatomical and physiological disposition for the condition (2). However, the etiological cause of the focal melanization remains unknown.

The black spots are primarily observed at slaughter of seawater farmed Atlantic salmon (3), and there are no reports that such spots are common in wild fish. Histologically, black spots appear heterogenous. The more severe black spots are classically characterized as chronic inflammatory reactions of granulomatous character, where macrophages are the dominating cell type, and the presence of melano‐macrophages gives the black discoloration (3). In a longitudinal study where the presence of spots was followed through the seawater production phase in a commercial farmed salmon population, it was concluded that red spots preceded the formation of black spots (2). The term red spots refer to foci in the white muscle assumed to be intramuscular hemorrhages. The red spots were found to have a stable low prevalence in the production period, while the black spots accumulated over time in the fish population in seawater (2). Histopathological classification of the melanized spots show that they develop over the time the fish population has spent in sea water, and the most serious granulomatous inflammatory changes appear a few months before slaughter and are associated with *Piscine orthoreovirus* 1 (PRV-1) (2). Aggregation of macrophages and other immune cells forming granulomatous structures in the black spots indicate a long-term activation of the immune response (4).

Both immunohistochemistry and *in situ* hybridization methods have demonstrated presence of PRV-1 in melanized foci (2, 4). PRV-1 is a very common infection in farmed Atlantic salmon in the marine phase (5). The presence of PRV-1 in the black spots has been associated with the severity of the spots (2, 4). However, due to the increasing prevalence of PRV-1 infection in farmed Atlantic salmon with time spent in seawater, an alternative hypothesis would be that the presence of melanized changes is coincidental and not caused by PRV-1 infection. In line with this, some macroscopic dark spots are found in fish without detectable PRV-1 infection, but histologically these spots do not show the same chronic inflammatory and granulomatous reactions (2). In black spots with histopathological changes, classified as granulomatous changes, PRV-1 seems to be a consistent finding (2).

PRV virions are naked particles of 70 nm-large icosahedral capsids encompassing the genome of ten double stranded (ds)RNA segments, categorized into long (L1-L3), medium (M1-M3) and small (S1-S4) segments. There are three recognized subtypes of PRV. PRV-1 is mainly found in Atlantic salmon where it may cause heart and skeletal muscle inflammation (HSMI) (6). Following infection of PRV-1 in Atlantic salmon, the virus replicates to high titers in its main target cell, the erythrocyte (7, 8), and subsequently high virulent variants of PRV-1 infect cardiomyocytes leading to the cardiac inflammation observed during heart and skeletal muscle inflammation (HSMI) (9).

Previous studies have indicated that Atlantic salmon does not clear the PRV-1 infection, and the acute infection develops into a persistent, low productive infection (10). In the persistent phase, PRV-1 infection can be found in circulating erythrocytes and renal erythroid progenitor cells, but also in macrophages and melano-macrophages in kidney and spleen (11). In the melanized spots and in the granulomatous reactions of the more severe black spots in particular, PRV-1 is found in macrophage-like cells, melano-macrophages and erythrocytes (2, 12). This could indicate that the infected cells have a role in the pathogenesis of the melanized changes. Melano-macrophages primarily reside in the spleen and head kidney of teleost fish, where they can cluster to form so-called melano-macrophage centers, but they may also migrate to inflamed tissues (13).

Macrophages are often classified according to their polarization rather than their tissue location. The M1 type macrophages are classically activated and polarized by IFN-γ signaling. They produce a pro-inflammatory microenvironment by secreting inflammatory cytokines, and have the capacity to inactivate intracellular pathogens through, among other factors, the action of nitric oxide (NO) and reactive oxygen species (ROS) (14, 15). Presence of M1 macrophages in an area with infection suggests that macrophage polarization have occurred through sensing of danger signals (16, 17). M1 macrophages are a common phenotype of phagocytes during a cell mediated immune response (18).

The M2 macrophages, on the other hand, are anti-inflammatory and are central in wound healing and tissue repair (19, 20). M2 macrophages can be activated by anti-inflammatory cytokines (IL-4 or IL-13) (21) and their main functions are to generate extracellular matrix and polyamines for cell growth and division, in addition to protein synthesis necessary for the healing process (22). There are many indications that the polarized macrophage phenotypes exist also in teleost fish (23, 24), and the presence of inducible nitric oxide synthase (iNOS2) and arginase-2 (Arg2) have been defined as M1 and M2 specific markers, respectively (22).

Interaction between cytotoxic T-lymphocytes (CTLs) and the antigen presenting complex MHC-I on the target cell surface can initiate the killing of target cells by the actions of granzymes and perforins produced by CTLs (25, 26). Involvement of CTLs in the host defense mechanism against PRV-1 infected cells is indicated in HSMI (27) and spots development (12). The specific co-localization pattern of PRV-1 and the targeted response of these immune cells can be exposed through multiplex *in situ* hybridization method.

This study aimed to characterize the polarization of macrophages in red and black spots by multiplex fluorescent *in situ* hybridization (FISH) method and to study the correlation of markers of macrophage polarization, MHC-I and CD8 expressing cells with PRV-1 infection. The reduction of the relative number of PRV-1 infected cells through the spots’ stages indicated an elimination of PRV-1 infected cells in the melanized focal spots in Atlantic salmon. Transformation of red spots into black spots is associated with the emergence of melano-macrophages of M2 phenotype in the white skeletal muscle.

## Material and Methods

### Samples From Field Trial

Atlantic salmon smolts with an average weight of 110 g were transferred to sea in a commercial setting at Svåsand, Hardanger, Norway, as earlier described (2). The fish were sampled regularly throughout the seawater period and screened visually for presence of red and black spots in the white muscle (2). Formalin fixed samples of red and black spots had been categorized and graded based on macroscopic appearance and the PRV-1 infection status of the population had been monitored by RT-qPCR of gill, spleen and muscle samples by PatoGen Analyse, Ålesund, Norway as earlier described (2). The population was PRV negative upon transfer to sea and the first PRV positive fish were detected at 23 weeks post transfer. At 48 weeks post transfer about 98% of the sampled fish were PRV-1 positive. The samples used in the present study were collected at 4 and 52 weeks post sea transfer, i.e. prior to PRV-1 infection and after the population was near completely infected with PRV-1, in this context referred to as PRV negative and positive, respectively. The seawater temperature was 11-11.5°C at samplings.

The samples were collected from white muscle of the cranio-ventral part of the fillet and were no spots (normal tissue), macroscopic red spots and black spots (**Table 1**). Macroscopically the spots were graded 1-3 where grade 1 was very faint discoloration, 2 was a distinct but not severe discoloration and 3 was a prominent and severe discoloration (2).

**Table 1:** Samples selected from red and black spots.

Category	PRV status	Grading	Sampling time (weeks after transfer to sea)
**Black spot**	Positive (n = 6)	Grade 1-3 black spots	52
	Negative (n = 6)	Grade 1-2 black spots	4
**Red spot**	Positive (n = 5)	Grade 1-3 red spots	52
	Negative (n = 6)	Grade 1-3 red spots	4
**No spot**	Positive (n = 6)	No macroscopic lesion	52
	Negative (n = 4)	No macroscopic lesion	4

### RNA Extraction and RT-qPCR

Total RNA was extracted from a 25 mg sample of the tissues from all fish from each group as shown in **Table 1** using 0.65 ml QIAzol Lysis reagent (Qiagen, Hilden, Germany). Tissues were homogenized using 5 mm steel beads in a TissueLyzer II (Qiagen) for 2 x 5 min at 25 Hz. Chloroform was added and the aqueous phase collected for automatic RNA isolation using a RNeasy Mini QIAcube Kit (Qiagen), eluting RNA in 50 µl RNase free water. RNA concentrations were determined in a Nanodrop ND-100 spectrophotometer (Thermo Fisher Scientific, Waltham, MA, USA). Thereafter, RNA was stored at -80°C until further use.

cDNA was synthesized from 1 µg total RNA by using Quantitect Reverse Transcription Kit (Qiagen) according to the manufacturer’s guidelines. In short, the procedure included elimination of genomic DNA and incubation at 42°C for 30 min with RT mastermix including reverse transcriptase enzyme and RNAse inhibitor. Quantitative PCR was performed in duplicates in 96-well plates, using a reaction volume of 12 µl with 15 ng cDNA input per well, and the Maxima SYBR Green/ROX qPCR Master Mix-2x (Thermo Fisher Scientific). Thermal conditions were set for an initial denaturation at 10 min/95°C and 40 cycles of amplification at 15 sec/95°C, 30 sec/60°C and 30 sec/72°C. Melting curve analysis were included to ensure assay specificity. Elongation factor (EF1ab) was used as reference gene (28). No-template control (NTC) were run on the same plate as negative control. The cut off value was set to Ct 35, and fold induction of genes of interest was determined against the reference gene and control samples (29). Primers (**Table 2**) were designed using Vector NTI Advance™ 11 software (Thermo Fisher Scientific), and AlignX application (Vector NTI Advance™ 11 Package, Invitrogen Dynal AS) was used for sequence alignments. (**Table 2**)

**Table 2:** List of specific primers for genes of interest.

Genes	Primer	Conc.	Sequence (5’-3’)	Amplicon (bp)	Accession No.
iNOS2*	F	400 nM	CATCGGCAGGATTCAGTGGTCCAAT	135	XM_014214975.1
	R		GGTAATCGCAGACCTTAGGTTTCCTC		
Arg2*	F	400 nM	CCTGAAGGACTTGGGTGTCCAGTA	109	XM_014190234.1
	R		CCGCTGCTTCCTTGACAAGAGGT		
**MHC Class I (** 30 **)**	F	400 nM	CTGCATTGAGTGGCTGAAGA	175	AF504022
	R		GGTGATCTTGTCCGTCTTTC		
**CD8α (** 31 **)**	F	400 nM	CACTGAGAGAGACGGAAGACG	174	AY693393
	R		TTCAAAAACCTGCCATAAAGC		
**Granzyme A (** 31 **)**	F	400 nM	GACATCATGCTGCTGAAGTTG	81	BT048013
	R		TGCCACAGGGACAGGTAACG		
**EF1αb (** 28 **)**	F	500 nM	TGCCCCTCCAGGATGTCTAC	57	BG933897
	R		CACGGCCCACAGGTACTG		

*Amplification efficiency (E) of newly designed primers were calculated for iNOS2 (E = 0.98) and Arg2 (E = 1.02).

### Statistical Analysis

Fold change (2- ΔΔCt formula) medians for genes of interest were compared in all groups, using non-parametric Mann-Whitney test due to small sample number, to display differences among the groups. GraphPad Prism version 9.0 (GraphPad Software Inc., La Jolla, CA, USA) was used for statistical analysis and graphical layouts. p ≤ 0.05 was considered as significantly different.

### Histology

Samples for histological examination were selected from PRV-1 infected and uninfected fish populations with or without macroscopic red and black spots (**Table 1**). Selection criteria for the uninfected population with red and black spots was spot grade level 2 (n = 1) because no uninfected fish had grade 3 level black spots. Samples from PRV-1 infected fish with red and black spots had grade 3 (n = 2). Samples from uninfected fish without macroscopic lesion were selected randomly and used as negative control, whereas samples from infected fish without spot were selected based on highest PRV-1 level (**Table S1**). Formalin fixed paraffin embedded (FFPE) tissue section (2 µm thickness) was dehydrated by gradual ethanol baths followed by xylene washing for paraffin clearance. Rehydration of the sections were performed for subsequent staining with hematoxylin and eosin (H&E staining). Standard procedures were followed (32). Bright field microscopy (Carl Zeiss Light Microscopy System with Axio Imager 2 - Carl Zeiss AG, Oberkochen, Germany) was performed for imaging.

### Fluorescent *In Situ* Hybridization (FISH)

#### Sample Pretreatment

FFPE sections were sliced with 5 µm thickness from tissue samples and mounted on Superfrost plus (Thermo Fisher Scientific) slides. Slides were baked at 60°C for 2 h in a HybEZ™ II oven (Advanced Cell Diagnostics, catalog #321720) followed by deparaffinization with 100% ethanol and fresh xylene baths. Samples were pretreated with hydrogen peroxide for 10 min at RT, boiled with RNAscope antigen retrieval reagent (Advanced Cell Diagnostics, catalog #322000) for 15 min at 99°C, and then incubated with RNAscope protease plus reagent for 15 min at 40°C in the HybEZ™ II oven. Hydrophobic barrier was made around the tissue section using Immedge hydrophobic barrier pen (Vector Laboratories, Burlingame, CA).

#### Multiplex *In Situ* Probe Hybridization

RNAscope^®^ Multiplex fluorescent V2 assay kit (Advanced Cell Diagnostics catalog #323100) was used for simultaneous detection of up to three different RNA targets. Probes (**Table 3**) were designed against; PRV-1 L3 segment (Advanced Cell Diagnostics catalog #537451) iNOS2 (Advanced Cell Diagnostics catalog #548391); Arg2 (Advanced Cell Diagnostics catalog #548381) CD8α (Advanced Cell Diagnostics catalog #836821); Granzyme A (Advanced Cell Diagnostics catalog #836841) and MHC-I (Advanced Cell Diagnostics catalog #836831). Probes against Peptidylpropyl Isomerase B (PPIB) (Advanced Cell Diagnostics, catalog #494421) was used as reference gene for RNA integrity of the target samples. Dihydrodipicolinate reductase (DapB), a bacterial gene from *Bacillus subtilis* (Advanced Cell Diagnostics catalog #310043) was used as negative control gene to assess cross-reactivity and background noise. Probes were mixed and hybridized for 2 hrs at 40°C in the HybEZ™ II oven. Amplification steps (Amp1-Amp3) were performed according to the manufacturer’s protocol. Opal fluorophores (**Table 2**) (Akoya Biosciences, CA, United States) were prepared and diluted (1:1500) using tyramide signal amplification (TSA) buffer (Advanced Cell Diagnostics catalog #322809) provided in the kit. Each probe was assigned a fluorophore, having a different emission and excitation range to distinguish each output signal (**Table 3**). Also, every probe was developed, labeled, and blocked separately by incubating with RNAscope^®^ multiplex Fluorescent Detection Reagents v2 (catalog #323110) and diluted Opal fluorophores in a sequential order as per manufacturer recommendations. Each section was counter stained by adding DAPI (fluorescent DNA stain) for 30 sec at room temperature. Mounting was performed by adding 1-2 drops of Prolong Gold antifade mounting reagent (Thermo Fisher Scientific). Imaging was performed in a TCS SP8 gSTED confocal microscope (Leica microsystems GmbH, Mannheim, Germany).

**Table 3:** List of probes and corresponding fluorophores used in FISH.

	Probe	Target Region (bp)	Fluorophores	Emission/Excitation Wavelength (nm)	Channel*
**Target**	PRV-L3	415–1379	Opal 520 (FP1487001KT)	494/525	C1
	iNOS2	2–949	Opal 620 (FP1495001KT)	588/616	C2
	Arg2	1332–2053	Opal 690 (FP1497001KT)	676/694	C3
	CD8α	8-1033	Opal 620 (FP1495001KT)	588/616	C2
	GzmA	3-1088	Opal 690 (FP1497001KT)	676/694	C3
	MHC-I	2-2321	Opal 620 (FP1495001KT)	588/616	C2
**Control**	PPIB	20–934	Opal 520 (FP1487001KT)	494/525	C1
	DapB	414–862	Opal 520 (FP1487001KT)	494/525	C1

*Channels signify the specific labeling of each fluorophore separately for their excitation and emission properties.

Accession numbers; PRV-L3- KY429945; PPIB- NM_001140870; DapB- EF191515, for the other genes the acc. nos. are listed in **Table 2**.

## Results

### Histology of Red and Black Focal Changes

**Figure 1:**
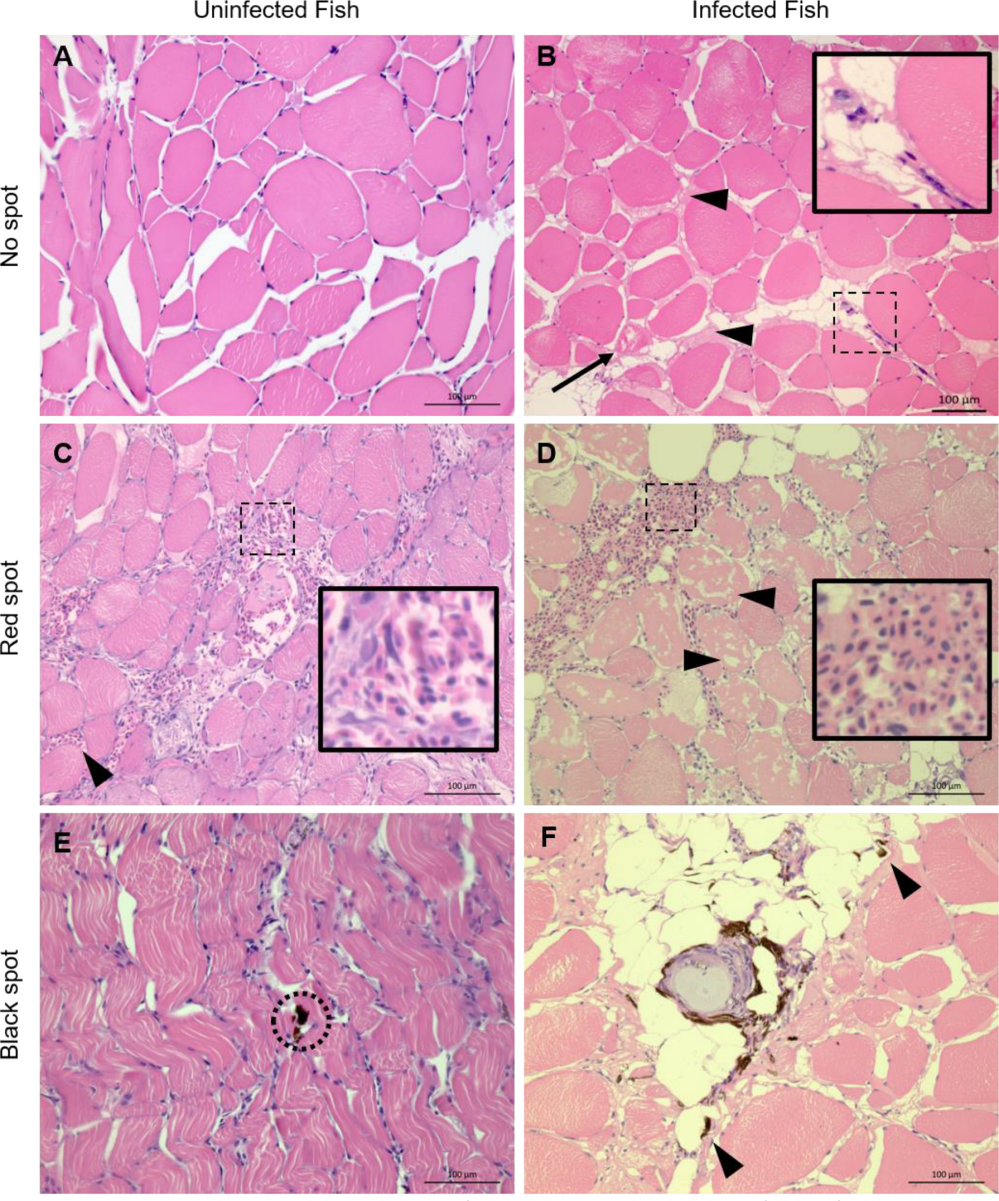
Representative histological sections from white skeletal muscle of noninfected and PRV-1 infected Atlantic salmon HE stained. **(A)** Myocytes with no observable changes. **(B)** Myocytes with mild degeneration (arrow) and presence of inter-myocytial fluid (arrowheads) and few infiltrating leukocytes (inset). **(C)** Uninfected fish, red focal change shows areas with moderate (inset) and minor (arrowhead) hemorrhages. Note minor degeneration in some myocytes with infiltrating macrophages. **(D)** Red focal change in PRV-1 infected fish with large hemorrhage (inset) and massive myocyte necrosis (arrowheads). **(E)** Black spot in uninfected fish. Sporadic occurrence of melano-macrophages (black) in seemingly otherwise non-affected tissue (dotted circle). **(F)** Black spot in infected fish with a typical granulomatous change surrounded with melano-macrophages (black). Arrowheads point to scattered melano-macrophages. Scale bar = 100 µm.

In samples from white muscle from non-infected fish without visible spots, unaltered and intact myocytes were seen (**Figure 1A**). In samples of non-spot tissue from PRV-1 infected fish, mild myocyte degeneration was observed to some extent along with presence of infiltrating leukocytes (**Figure 1B**).

In red spots the white skeletal muscle tissue showed moderate to severe bleedings, and mild degeneration to moderate myocyte necrosis was observed in uninfected and infected fish, respectively (**Figures 1C, D**). The red spots from PRV-1 infected fish differed from the noninfected fish by infiltration of leukocytes and scattered appearance of adipocytes. In black spots, melanin was found in both groups (**Figures 1E, F**), however, the presence of macrophage-like cells with histologically observable melanin content, referred to as a melano-macrophages, in the samples of the infected fish were more prominent and widespread. Moreover, granulomatous changes in the black spots were observed in PRV-1 positive fish (**Figure 1F**) and never in the uninfected fish.

### *In Situ* Localization of Differentiated, Polarized Macrophages and PRV-1 in Focal Changes

**Figure 2:**
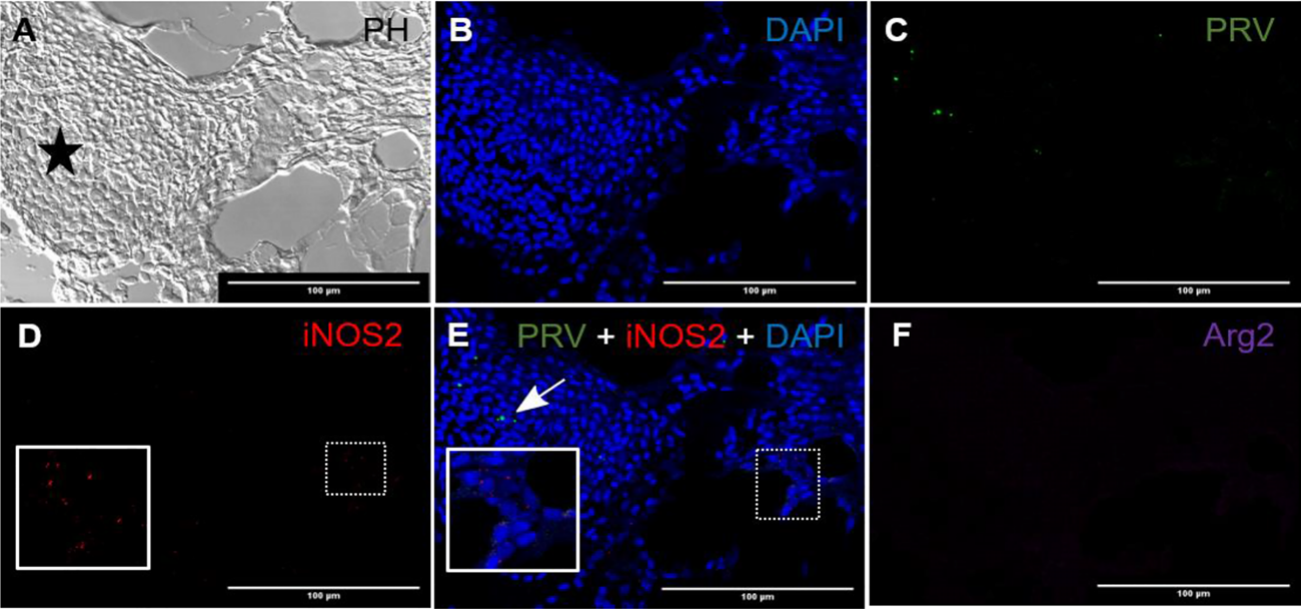
*Fluorescent in situ hybridization of PRV-1, iNOS2 and Arg2 in red focal changes (early phase)*. **(A)** Phase contrast image showing a large hemorrhage (star). **(B)** DNA staining of the cells by DAPI (blue). **(C)** Presence of a few PRV-1 (green) positive cells in the hemorrhage. **(D)** iNOS2 (red) specific transcripts detected in a limited number of cells surrounding a peripheral blood vessel. **(E)** Merged image showing presence of PRV-1 (arrow) but no co-localizing in the M1 macrophage (inset). **(F)** Arg2 (purple) specific transcripts (M2 type macrophages) were undetected. Scale bar = 100µm.

**Figure 3:**
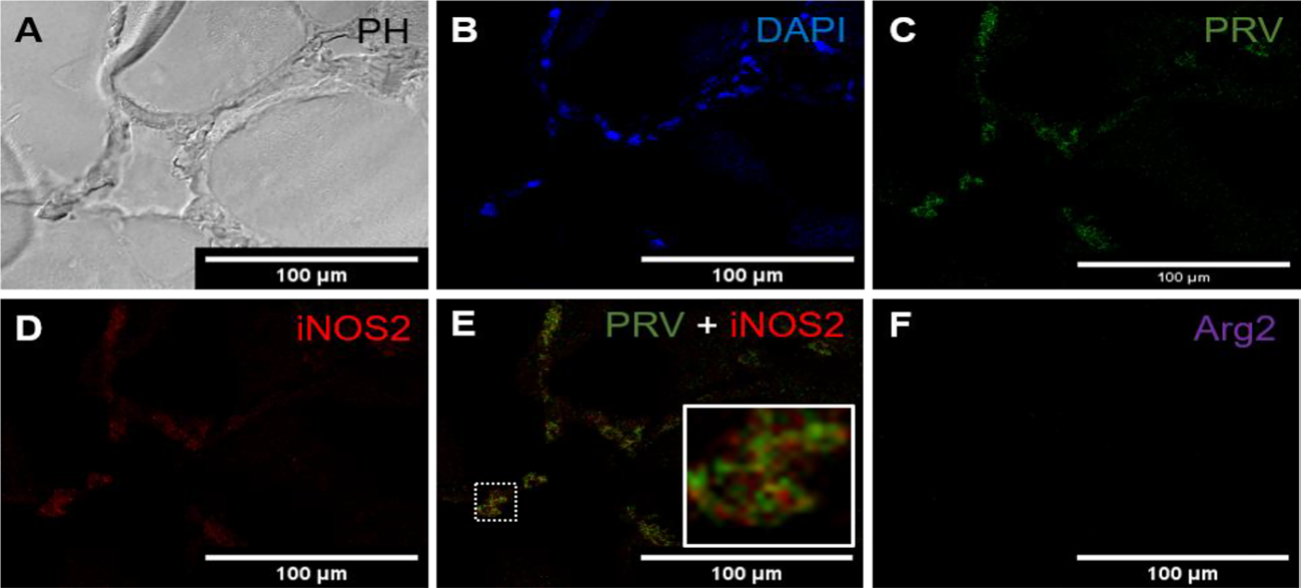
*Fluorescent in situ hybridization of PRV-1, iNOS2 and Arg2 in red focal changes (intermediate phase).* **(A)** Phase contrast image showing infiltrating cells between myocytes. **(B)** Nuclei DNA staining of the cells with DAPI (blue). **(C)** Presence of PRV-1 (green) in infiltrating cells in between myocytes. **(D)** Presence of iNOS2 (red) in infiltrating cells between myocytes. **(E)** Merged image showing co-localization (inset) of PRV-1 and iNOS2 (yellow). **(F)** Arg2 transcripts (purple) were not detected. Scale bar = 100µm.

**Figure 4:**
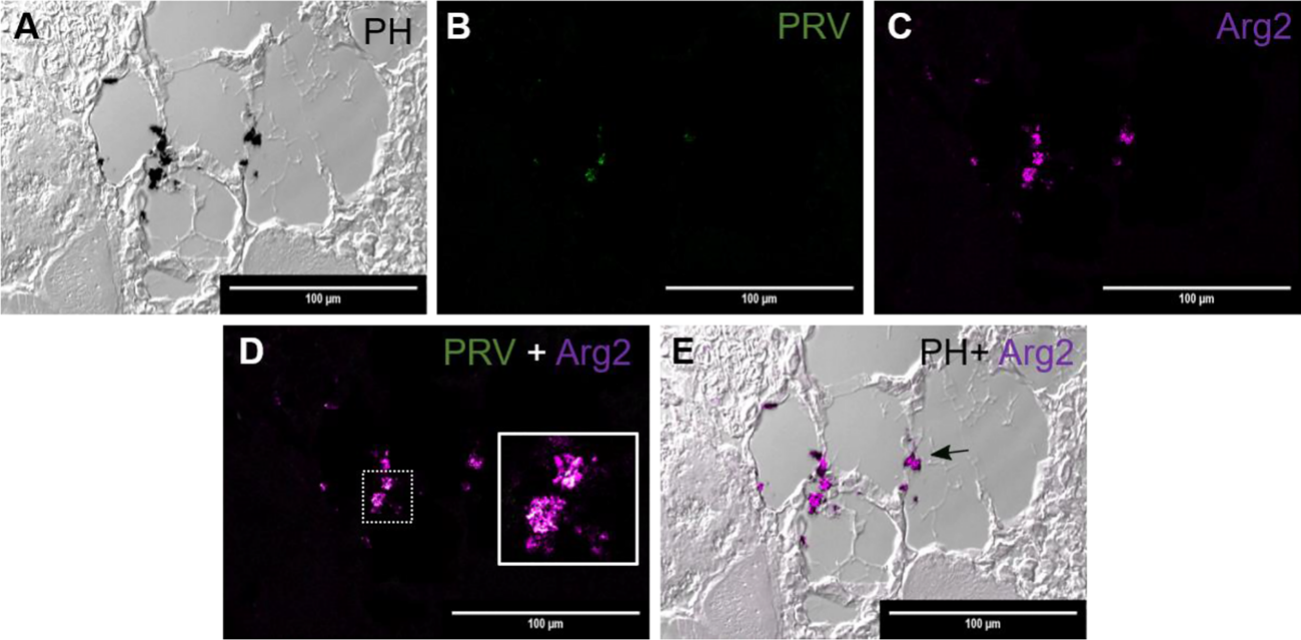
*Fluorescent in situ hybridization of PRV-1 and Arg2 in red focal changes (late phase).* **(A)** Phase contrast image showing structure of the analyzed area and melanin deposit. **(B)** Sporadic presence of PRV-1 (green) in melanized area. **(C)** Arg2 (purple) positive cells **(D)** Merged image showing PRV-1 and Arg2 co-localization (white in the inset). **(E)** Merged image showing Arg2 positive staining of melano-macrophages (arrow). Scale bar = 100µm.

**Figure 5:**
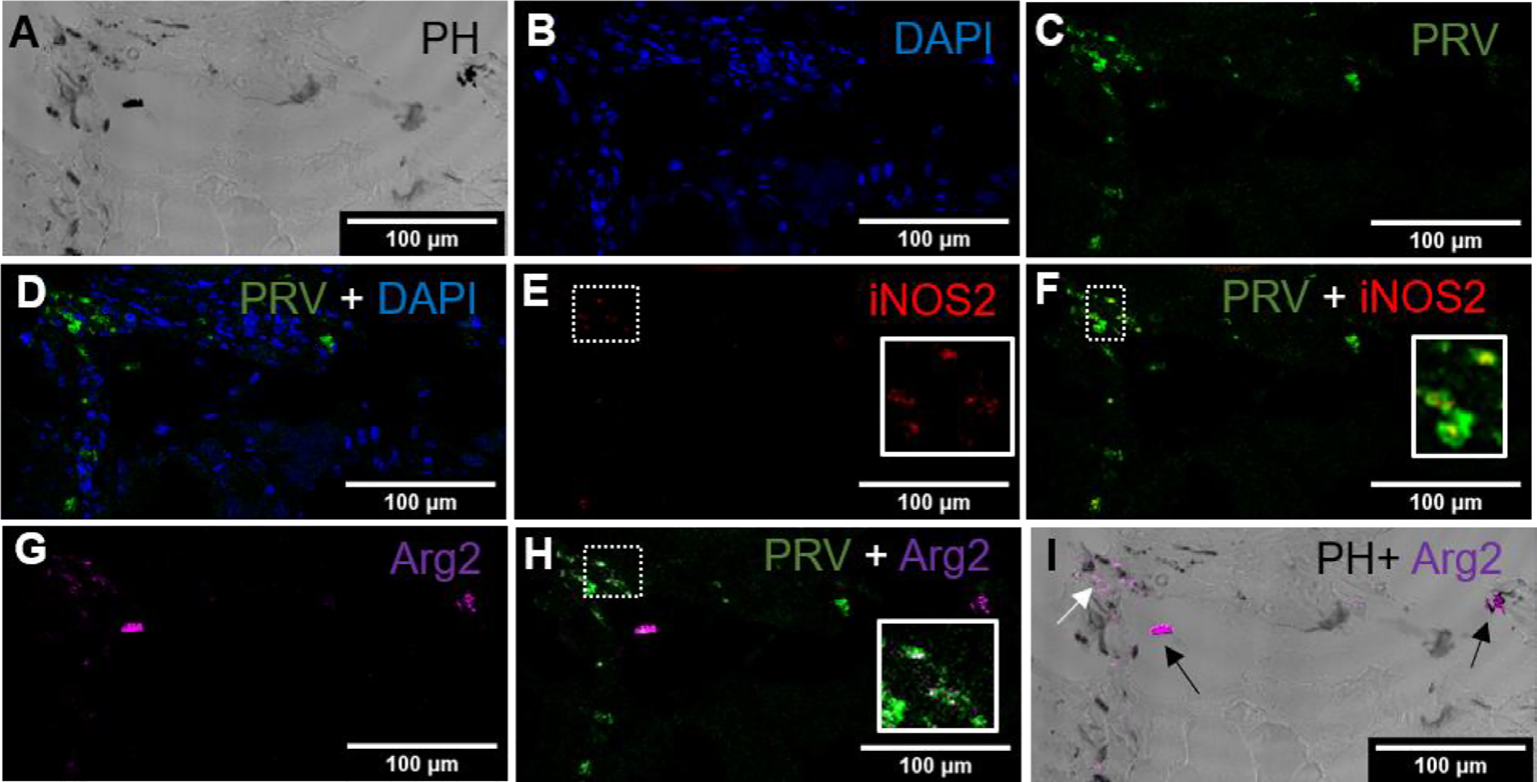
*Fluorescent in situ hybridization of PRV-1, iNOS2 and Arg2 in black focal changes (late phase).* **(A)** Phase contrast image presence of melanin in the infected area. **(B)** Nuclei DNA stained with DAPI (blue). **(C)** PRV-1 (green) detected in severe melanized area **(D)** Merged PRV-1 and DAPI. Number of PRV-1 positive cells compared to total number of cells were low. **(E)** Few iNOS2 (red) positive cells detected at the infected area **(F)** Merged image showing co-localization (yellow in inset) of PRV-1 and iNOS2. **(G)** Presence of Arg2 (purple) positive cells. **(H)** Co-localization of PRV-1 and Arg2 positive cells (white in inset). **(I)** Localization of Arg2 specific transcripts in melanized M2 melano-macrophages (black arrows) and non-melanized M2 macrophages (white arrow). Scale bar = 100µm.

**Figure 6:**
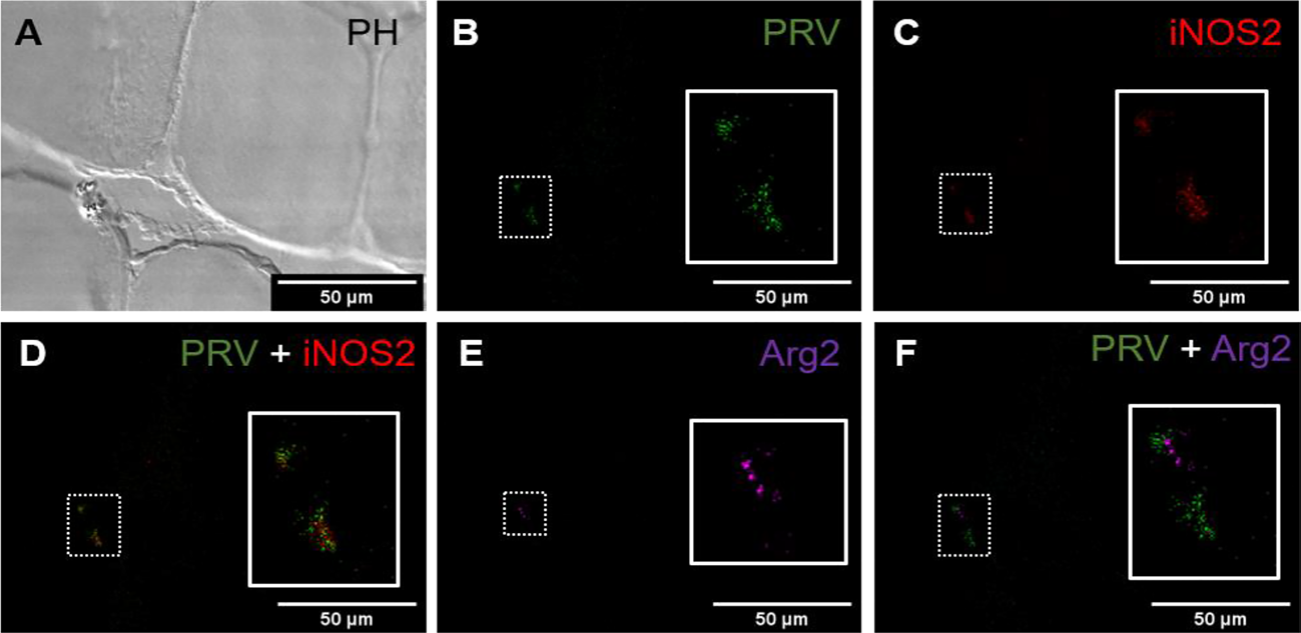
*Fluorescent in situ hybridization of PRV-1, iNOS2 and Arg2 in areas without spots, from PRV-1 infected fish.* **(A)** Phase contrast image showing cells structure. **(B)** PRV-1 (green) specific transcripts detected between muscle cells. **(C)** iNOS2 (red) positive cells in the same area as PRV-1. **(D)** Merged image showing co-localization of PRV-1 and iNOS2 (yellow, insert). **(E)** Arg2 (purple) positive cells were detected partly in same area as PRV-1. **(F)** Merged image of PRV-1 and Arg2 show Arg2 positive cells surrounding PRV-1 infected cells (inset). Scale bar = 50µm.

*a. Uninfected fish*
The positive and negative controls, i.e. using the PPIB and DapB probes, are shown in **Figures S1**, **S2**, respectively. Tissues with macroscopic appearance of red focal changes from uninfected fish showed no iNOS2 specific staining, but some Arg2 positive cells (**Figure S3**). Similarly, sections with the macroscopic appearance of black spots from uninfected fish showed low number of iNOS2 or Arg2 positive cells (**Figure S4**). No staining was seen in areas without spots from uninfected fish (**Figure S5**). No PRV-1 signal was detected from noninfected fish groups having spots or no spots (**Figures S3**–**S5**).
*b. Red spot. Early phase. PRV-1 infected.*
In red focal changes there were hemorrhages (**Figure 2A**) containing a large number of nucleated cells (**Figure 2B**). Hemorrhages analyzed by FISH showed PRV-1 in a few erythrocytes (**Figure 2C**). Numerous iNOS2 positive macrophages, i.e. M1 type polarized macrophages, were surrounding the hemorrhage (**Figure 2D**), but co-localization of PRV-1 and iNOS2 were not seen (**Figure 2E**). There was no staining for Arg2, i.e. M2 type polarized macrophages (**Figure 2F**). Due to the low presence of M1 activated macrophages and lack of organization of the hemorrhages, this appearance was assessed as being an early phase of the red spots.
*c. Red spot. Intermediate phase. PRV-1 infected.*
In red focal changes from infected fish, where the changes were assessed as more advanced and infiltrating cells were seen between the myocytes (**Figure 3A**). The large number of extravasal erythrocytes of the early phase was not present (**Figure 3B**). Co-localization of PRV-1 and iNOS2 was observed among infiltrating cells found between myocytes (**Figures 3C, D**). There was no staining with Arg2 (**Figure 3F**). Due to the high presence of M1 activated macrophages and the organized appearance of the hemorrhages, but lack of melano-macrophages, this was assessed as being an intermediate phase of the red spots.
*d. Red spot. Late phase (transition between red and black spots). PRV-1 infected.*
In another region from the same red spot sample, as displayed in **Figure 3**, there were some scattered deposits of melanin (**Figure 4A**). In these areas, there was a modest number of PRV-1 positive cells (**Figure 4B**.) Here, Arg2 specific transcripts were detected (**Figure 4C**), with co-localized PRV-1 staining (**Figure 4D**). Detection of Arg2 was only observed in melano-macrophages found in the sporadic melanin deposits (**Figure 4E**). The commencement of M2 type melano-macrophage detection was assessed as an indication of transition from red to black spots.
*e. Black spots. PRV-1 infected*
In macroscopic black spots, larger deposits of melanin were seen (**Figure 5A**), and there was a moderate density of cells (**Figure 5B**). PRV-1 stained cells were mainly seen in the area of melanization (**Figures 5C, D**). Scattered areas of iNOS2 positive cell populations were detected in the melanized focal changes (**Figure 5E**), but these only partly co-localized with PRV-1 staining (**Figure 5F**). Arg2 positive cells were also seen in the PRV-1 infected area together with melanin presence (**Figure 5G**), showing some co-localization of PRV-1 and Arg2 (**Figure 5H**). A number of melano-macrophages with PRV-1 were detected. Arg2 positive transcripts were primarily detected in melano-macrophages, but were also present in non-melanized M2 macrophages (**Figure 5I**).
*f. No focal changes, PRV-1 infected*
In PRV-1 infected fish, samples from areas in white muscle without spots showed co-localization of iNOS2 and PRV-1 (**Figures 6A–D**), while Arg2 and PRV-1 only partly overlapped (**Figures 6E, F**). The staining of iNOS2 and Arg2 did not overlap.

### Presence of CD8^+^ and MHC-1 Positive Cells

**Figure 7:**
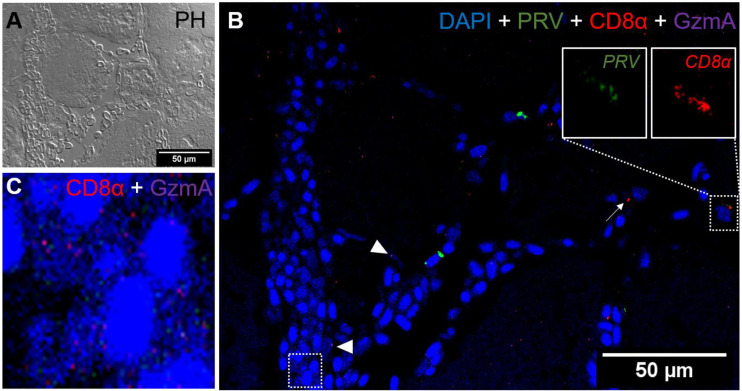
*Fluorescent in situ hybridization of PRV-1, CD8α and GzmA in red focal changes.* **(A)** Phase contrast image showing a bleeding area with a large aggregation of blood cells. **(B)** Merged image of PRV (green), CD8α (red) and GzmA (purple). Localization of PRV-1 in CD8+ cell (dotted rectangle at right top) and co-expression of granzyme A in CD8^+^ T cells (dotted rectangle in left bottom). Individual T cells detected expressing granzyme A specific transcripts (arrowhead) along with other CD8 cells (arrow). Nuclei DNA stained with DAPI (blue) **(C)** Magnified image of CD8+ and GzmA co-expression from dotted square in image **(B)** Scale Bar = 50 µm.

**Figure 8:**
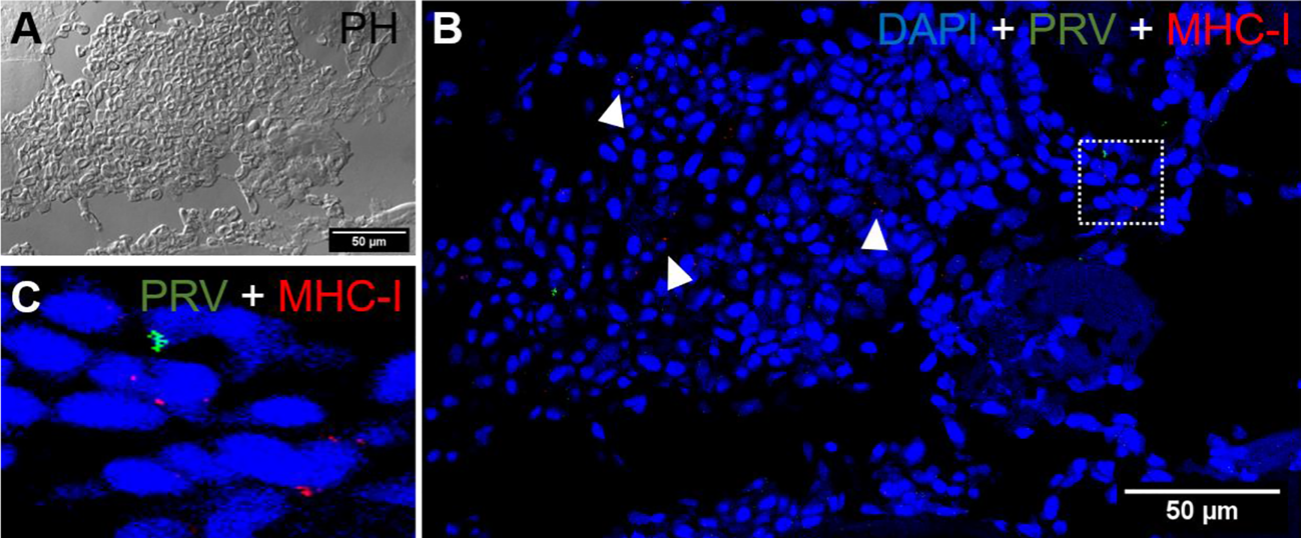
*Fluorescent in situ hybridization of PRV-1, and MHC-I in areas of red focal changes.* **(A)** Phase contrast image showing a large hemorrhage. **(B)** Merged image of PRV-1 and MHC-I showed no co-expression of PRV-1 in MHC-I cells, but a few cells were detected in the bleeding area (arrowhead). **(C)** Magnified image from image B (dotted rectangle) showing PRV-1 infected cells along with MHC-I cells. Scale Bar = 50 µm.

**Figure 9:**
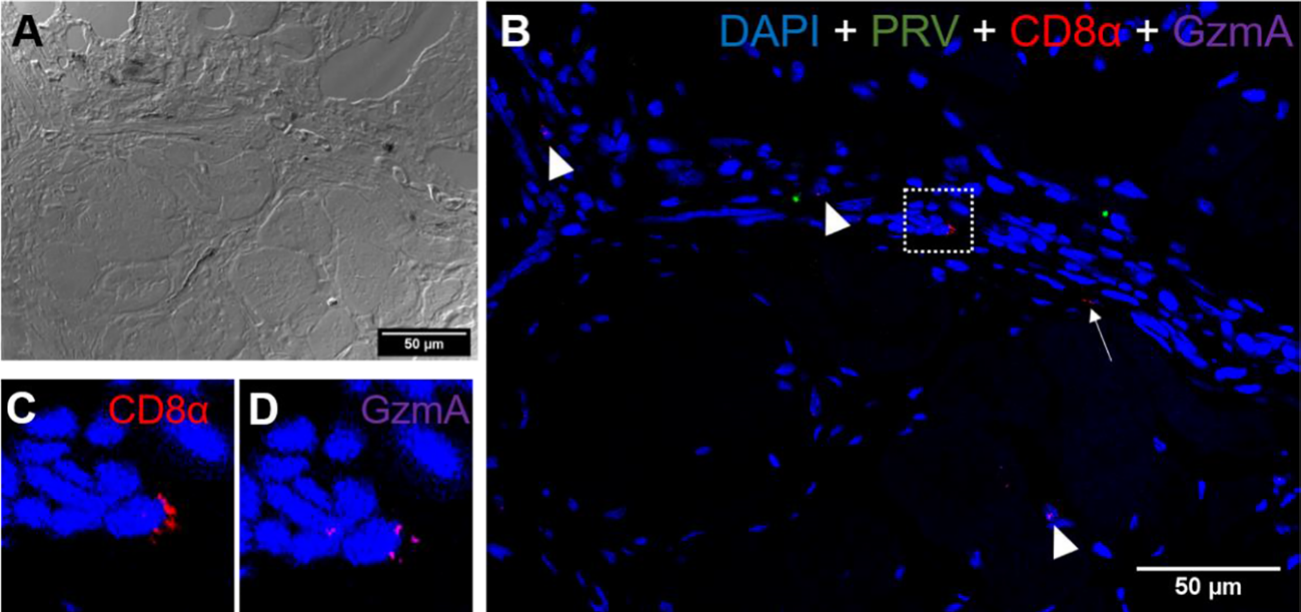
*Fluorescent in situ hybridization of PRV-1, CD8α and GzmA in black focal changes.* **(A)** phase contrast image showing cell structures with melanin accumulation. **(B)** Merged image showing presence of PRV-1 (green) infected cells with CD8^+^ cells (red) (arrows) and granzyme A (purple) in another cell population (arrowhead). Dotted rectangle showing co-expression of CD8 and GrzmA split in **(C, D)**. Nuclei DNA stained with DAPI (blue) Scale Bar = 50 µm.

**Figure 10:**
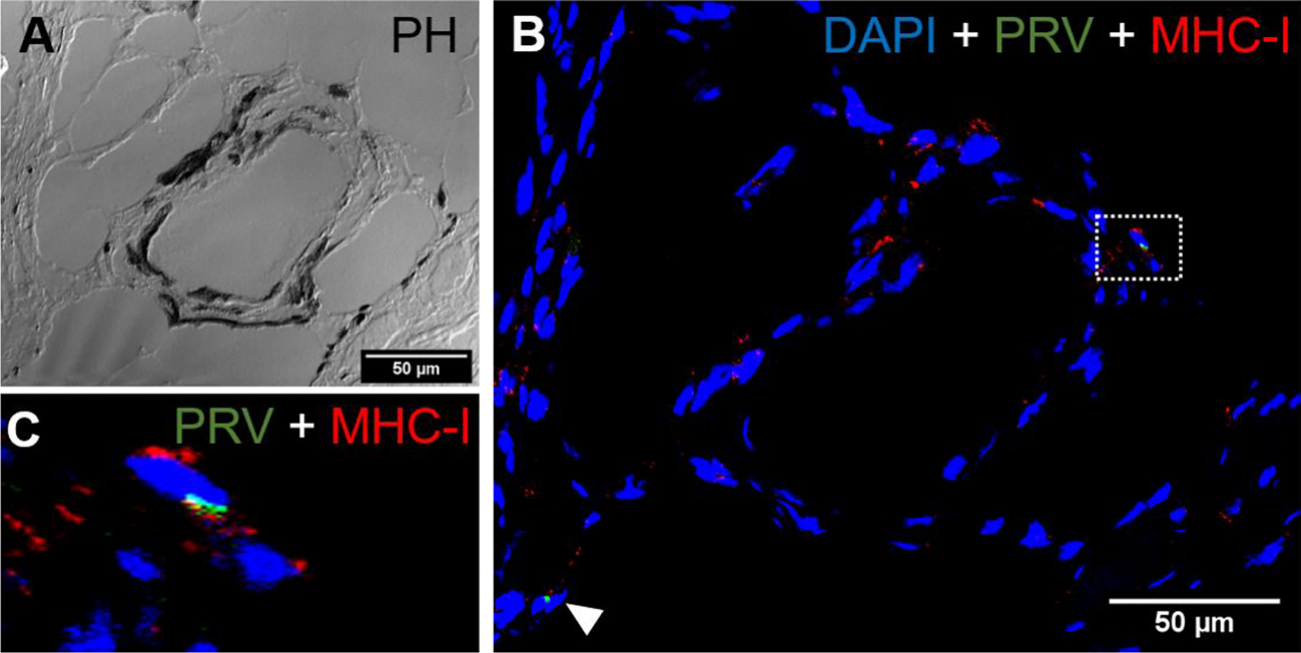
*Fluorescent in situ hybridization of PRV-1 and MHC-I in black focal changes*. **(A)** phase contrast image showing vacuolar area surrounded by melano-macrophages and other immune cells. **(B)** Merged image showing presence of numerous MHC-I positive cells (red) where some are co-staining with PRV-1 (green) (dotted rectangle and arrowhead). **(C)** Magnified area from image B showing co-localization of PRV-1 in some MHC-I cells. Scale Bar = 50 µm.

*a. Red Focal Changes, PRV-1 infected*
*In situ* labeling revealed a mild influx of CD8^+^ cytotoxic T lymphocytes (CTLs) in the bleeding area of red focal changes in PRV-1 infected fish (**Figure 7A**). Some of the CD8+ cells were also positive for PRV-1 staining, whereas other CD8-positive cells were present around infected cells (**Figures 7A, B**). Granzyme A transcripts were detected in both CD8^+^ (**Figures 7A–C**) and other cell populations in the infected area (**Figures 7A, B**). Numerous MHC-I positive cells were present at the bleeding site (**Figures 8A–C**), with a limited number also being PRV-1 infected. PRV-1 did not appear to co-localize with MHC-I (**Figures 8B, C**).
*b. Black Focal Changes, PRV-1 infected*
CD8+ cells were detected in the areas with melanin deposits. Some PRV-1 infected cells were also detected in this area, but the staining did not co-localize (**Figure 9**). Granzyme A specific transcripts were co-localized with CD8+ (**Figures 9C, D**) but also found in other cell subsets (**Figure 9B**). Numerous MHC-I positive cells were detected around a vacuolar area surrounded by melano-macrophages and some PRV-1 infected cells, showing high melanin deposits (**Figures 10A, B**). PRV-1 did co-localize with some MHC-I stained cells (**Figure 10C**).

### Gene Expression in Red and Black Spots

**Figure 11:**
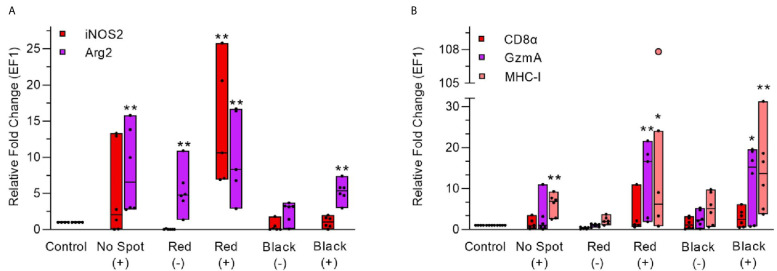
Gene expression analysis during red and black focal changes in PRV infected (+) and uninfected (-) fish groups. **(A)** Relative fold change (median line) for each fish group is shown for iNOS2 and Arg2 (left Y-axis). **(B)** Relative fold change (median line) for each fish group is shown for CD8α, GzmA and MHC-I. Pink dot showing outlier value in the respective group. Each dot within boxes represents individual fish in the group. Gene expression relative to the control group (uninfected and unaffected) fish was normalized against EF1ab. * indicates significantly different from the control group (*p < 0.05, **p < 0.01).

*a. iNOS2 and Arg2*
iNOS2 expression was low in PRV positive (median Ct 30.7), non-spot samples (2 folds)while it was significantly increased (approximately 10 folds, MWU value = 0, n1 = 4, n2 = 5, p-value = 0.0079) in PRV positive (median 27) red focal changes (**Figures 11A**, **S6**). In contrast, iNOS2 was not upregulated in the samples from red focal changes of uninfected fish. In the black focal changes, iNOS2 expression was at the same level as in non-spot samples. Arg2 expression was significantly upregulated in all of the target groups, especially in PRV-1 infected groups. Arg2 was upregulated both in infected, no spot samples (6.5 folds, MWU value = 0, n1 = 4, n2 = 6, p-value = 0.0048) and in red focal changes without PRV infection (4.7 folds, MWU value = 1, n1 = 4, n2 = 6, p-value = 0.0095), compared to the uninfected, no-spot control. Both iNOS2 and Arg2 expression were at the highest level in the PRV infected fish with red focal changes. But in black focal changes only Arg2 expression, in contrast to iNOS2, was significantly upregulated (MWU value = 0, n1 = 4, n2 = 6, p-value = 0.0048) in PRV-1 infected group (median 27.7) (**Figures 11A**, **S6**).
*b. CD8α, GzmA and MHC-I*
There was a trend towards upregulation of the CD8α gene in the red and black focal changes (**Figure 11B**) but this was not statistically significant. Granzyme A expression level was significantly upregulated in PRV-infected groups with red (16.5 folds, MWU value = 0, n1 = 4, n2 = 5, p-value = 0.0079) and black focal changes (approx. 15 folds, MWU value = 2, n1 = 4, n2 = 5, p-value = 0.0489). Non-infected groups showed no significant induction of CD8α and granzyme A. Increased expression of MHC-1 was spotted in all fish groups infected with PRV-1, compared to non-infected groups, and the MHC-I expression level was relatively higher in black focal changes (14 folds, MWU value = 0, n1 = 4, n2 = 6, p-value = 0.0048) than in red focal changes (9 folds, MWU value = 2, n1 = 4, n2 = 5, p-value = 0.0317) (**Figure 11B**).

## Discussion

This study aimed to clarify the role of PRV-1 infection and the immune mechanisms involved in the development of melanized foci in white muscle of Atlantic salmon, using immune cell gene markers representing the macrophage polarization pattern, and the cytotoxic immune response.

Macrophages respond to their environment by differentiating into the functional pro-inflammatory phenotypes M1 macrophages, implicated in initiating and sustaining inflammation, or the anti-inflammatory M2 macrophages, implicated in tissue repair (33). In samples from red and black focal changes from non-infected fish there were no obvious detection of macrophage polarization apart from minimal occurrence of M2 macrophages, based on Arg2 transcript detection. Unaffected muscle areas of non-infected fish showed no presence of polarized macrophage markers. These findings were also reflected in the qPCR transcript analysis, which mirrored the *in situ* findings.

On the other hand, our results indicate that in the PRV-1 infected fish, the initial phase of the progress of the red and black spot formation were tightly connected to macrophage polarization and linked to the presence of PRV-1. The development of melanized focal changes is considered to be multifactorial. Viral diseases such as pancreas disease (PD) may affect the white muscle, but widespread presence of melanized changes in areas being free from PD historically suggest no etiological role for this disease in the development of melanized focal changes (34). Furthermore, melanized changes have not been found to be influenced by bacterial components (2). We found that local PRV-1 infection was associated with the M1 polarized cell marker iNOS2 in the early developmental phases of melanized foci. PRV-1 was detected in a limited number of erythrocytes in hemorrhages, and only a few M1 macrophages was detected in the initial phase of red spots. Erythrocytes are a primary cell target of PRV-1 both in the acute and persistent phases of infection (7), and infected cells can be detected in any vascularized tissue. We have not found evidence here indicating that the PRV-1 infection initiated these hemorrhages as in previous studies (2, 4). However, for the close PRV relative, Grass carp reovirus (GCRV), it is suggested that iNOS2 activity is implicated in apoptosis of the vascular endothelial cells in hemorrhages characteristic for GCRV infection of Grass carp (*Ctenopharyngodon idella*) (35).

In PRV-1 infected fish, the M1 type macrophages were modestly detected in the early phase of red focal changes. However, in the more developed, intermediate phase of red spots the M1 macrophages were a dominating feature and were almost uniformly positive for PRV-1 specific staining, i.e. PRV-1 infected. The expression analysis by RT-qPCR also demonstrated elevated expression of iNOS2 in this phase. The dominating presence of PRV-1 infected, M1 polarized macrophages in this phase indicates a pro-inflammatory environment, which may be driven by the PRV-1 infection. In an earlier study, a significant downregulation of the anti-inflammatory cytokine IL10 was found associated with red changes (12), which indirectly indicates a pro-inflammatory environment.

Red spots with sporadic appearance of melano-macrophages were categorized as late red spot phase. This phase is considered to reflect the transition phase between red and black spots. Upregulation of iNOS2 level during red spots could be an indicator for commencement of melanogenesis. It is noteworthy that iNOS2 contributes to the melanogenesis in mammalian melanocytes (36). Based on the expression of the M2 marker Arg2, we found that the melano-macrophages at the site displayed the properties of anti-inflammatory M2 macrophages. We also found M2 macrophages without melanin. Co-localization pattern revealed PRV-1 abundance in melanized cells. The melano-macrophages of teleost fish are phagocytic cells (37) and they accumulate at long-term antigen retention sites in salmonids (13, 38). Phagocytosis of virus infected cells by macrophage and melano-macrophages have been reported earlier in Atlantic salmon (39). M2 macrophages are cells normally involved in tissue repair, and here they appeared first when melanin started to accumulate in the spots in skeletal muscle tissue.

Our findings indicate that PRV-1 infected macrophages are not innocent bystanders but represent M1 polarized macrophages important in the development of the pro-inflammatory microenvironment of red spots. The melanin accumulation starts in the late phase of red focal changes and will ultimately progress into black focal changes. It therefore seems as if melano-macrophages do not infiltrate the changes as such, but rather as non-pigmented macrophages capable of accumulating melanin over time. Melanogenesis has previously been demonstrated in advanced black spots (3). This putative progression could also be an explanation for the low prevalence of red spots but an increasing prevalence of black spots through the production period in seawater (2).

The black spots demonstrated a more heterogenous macrophage populations, i.e. both M1 and M2 macrophages were present. In advanced melanized areas, few M1 macrophages were positive for PRV-1, whereas PRV-1 co-localization was detected both in melanized (melano-macrophages) and non-melanized M2 type macrophages. In mammals, Arg2 is shown to downregulate the nitric oxide production of the M1 macrophages (40). Our findings indicate that Arg2 specific transcripts are mostly linked to the melanized area and associate with melano-macrophages. Presence of melano-macrophages (M2) was consistent from the late phase of red spots into black spots transformation (**Table 4**).

**Table 4:** Consolidated summary of results.

Type of spot		*Key In situ* findings	Characteristic gene expression level	
**Red spot**	Early	Few M1 macrophages in PRV-1 positive hemorrhages.	Significant upregulation of *iNOS2* expression	Significant upregulation of *MHC-I* and *GzmA* expression
	Intermediate	High co-localization of PRV-1 in M1 macrophages.		
	Late	Detection of few M2 melano-macrophages.		
**Black spot**		Domination of M2 melano-macrophages and co-localization with PRV-1.	Significant upregulation of *Arg2* transcription.	

The correlated upregulation of Arg2 transcripts with the stage of development of the spots in the PRV-1 infected fish indicated a gradual shift from an inflammatory to a healing response during the transition from red to black macroscopic appearance of the spots. The spots of the non-infected fish with lack of detection of M1 macrophage marker and only a few detected M2 polarized macrophages, strongly indicated that PRV-1 is driving macrophage polarization in the spots of infected fish.

The initial etiological cause(s) of the red spots is unknown. The outcome of the spots in uninfected fish groups is also unknown due to the ubiquitous presence of PRV-1 in farmed Atlantic salmon, and the lack of an experimental model for spot formation (11). However, it could be speculated if the lack of inflammation in spots in non-infected fish argues for a shorter longevity and lower severity of the spots.

To further characterize the inflammatory microenvironment in the spots, the presence of CD8, Granzyme A and MHC-I positive cells was characterized during spot development. There were substantial variations in the presence of these markers among the individual fish, but *in situ* visualization indicated that MHC-I positive, PRV-1 infected cells were targeted by CD8 positive T cells both in red and black spots. The relative low number of CD8 positive cells evenly observed both in red and black focal changes was in line with the RT-qPCR expression analysis. However, a moderate, but not statistically significant up-regulation of CD8α expression in black focal changes compared to red focal changes was observed, and has been reported earlier (12). Mature cytotoxic T cells can use granzyme A for killing of target cells containing intracellular pathogens. Here, granzyme A specific transcripts were observed in cells that were not expressing CD8. By RT-qPCR, expression of Granzyme A was found to be significantly increased in both red and black spots compared to control samples, while CD8 was not. Granzyme A is also be synthesized by natural killer cells (NK-cells) (41) or other immune and non-immune cells in the teleost fish (42).

Mammalian myopathies are often marked by up-regulation of MHC-I (43, 44). By immunolabelling, MHC-I positive cells have earlier been demonstrated to be abundant in red spots (12), and in the present *in situ* study MHC-I cells were common, but perhaps not abundant. In both studies an absence of MHC-I positive myocytes was observed in the affected area, combined with lack of observation of PRV-1. The present study did not indicate that infection of the skeletal muscle cells is an important factor of the spot formation. As for Granzyme A, the MHC-I expression was significantly increased in both PRV-1 infected red and black spots compared to control samples, and co-localization of MHC-I and PRV-1 were seen in some cells especially in the melanized areas. Taken together, the targeted cell mediated immune response by the host tries to resolve and eradicate PRV-1 infection during red and black spots formation.

## Conclusion

A possible course of events in the pathogenesis of black spots, is that PRV-1 infected erythrocytes in the hemorrhages infect tissue macrophages through phagocytosis. The myocyte degeneration in red muscle caused by PRV-1 (6), could be an additional driver for influx of macrophages, but is probably not the initial cause of red spots, as these are found at similar prevalence prior to PRV infection (2). The iNOS2 expressing M1-polarized non-melanized macrophages are mainly present in the period of the red focal changes, i.e. the time of inflammation, which suggests local production of NO and other oxygen radicals by the M1 macrophages. Melanin is a protector against free oxygen radicals (45), and its accumulation could be a consequence of the pro-inflammatory environment. Melanogenesis has previously been demonstrated in a salmon macrophage-like cell line (46, 47). The increased prevalence and severity of the black spots over time, indicates that the spot forming process is long lasting. The numerous M2-polarized melano-macrophages in black spots indicate that this is a healing phase of the process. Moreover, the presence of cytotoxic T cells and MHC-I positive cells in the focal changes represents the host’ ability to target and eliminate PRV-1 infected cells. This suggests a role of PRV-1 infection in driving the development of black spots in white muscle of Atlantic salmon.

## Data Availability Statement

The raw data supporting the conclusions of this article will be made available by the authors, without undue reservation.

## Ethics Statement

Ethical review and approval was not required for the animal study because the material was achieved from commercial production.

## Author Contributions

Conceptualization, MM, HB and ER. Methodology, MM, IN and HB. Software, MM. Validation, MM, ØW and MD. Formal analysis, MM, HB, ØW and ER. Investigation, MM, HB, ØW and ER. Resources, ER and EK. Data curation, MM, IN. Writing—original draft preparation, MM and ER. Writing—review and editing, ØW, MM, MD, HB, EK and ER. Visualization, MM, IN and HB. Supervision, ER, EK, MD and ØW. Project administration, ER. Funding acquisition, ER and EK. All authors contributed to the article and approved the submitted version.

## Funding

The study was supported by the Norwegian Seafood Research Fund (FHF) grant 901501, and by the Research Council of Norway, grant 280847/E40 (ViVaAct).

## Conflict of Interest

The authors declare that the research was conducted in the absence of any commercial or financial relationships that could be construed as a potential conflict of interest.
