# Supplementary material for: PRV-1 Infected Macrophages in Melanized Focal Changes in White Muscle of Atlantic Salmon (Salmo salar) Correlates With a Pro-Inflammatory Environment
Source: Front Immunol. 2021 Apr 29;12:664624. doi: 10.3389/fimmu.2021.664624 (PMC8116804; doi:10.3389/fimmu.2021.664624)
Supplement: Supplementary file 1 [file DataSheet_1.docx]

**Supplementary Figures**


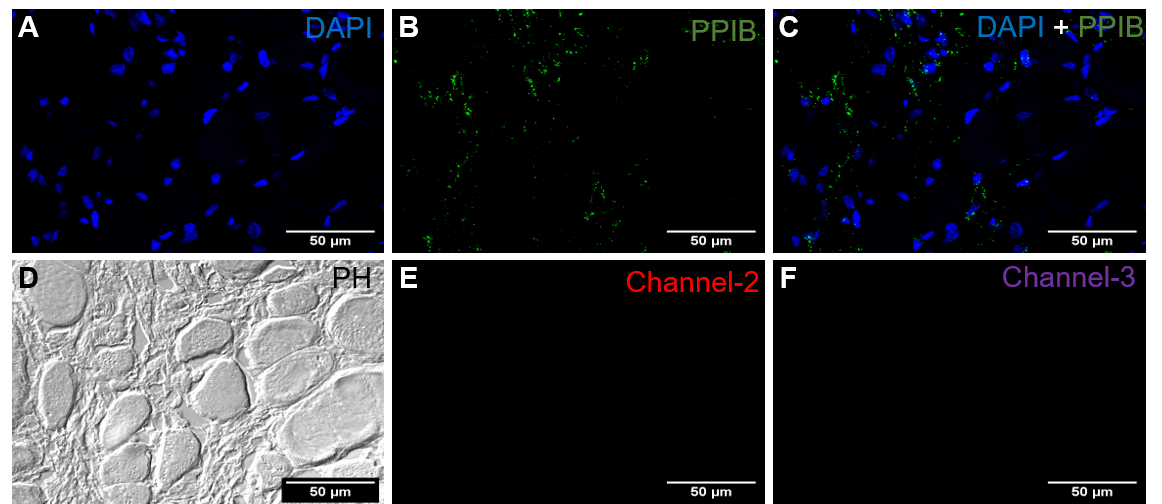
**Figure S1**. Positive Control for Fluorescent in situ hybridization (FISH) assay **(A+B+C)** Cellular nuclei DNA stained with DAPI. Homogenous and punctated expression of PPIB (green) specific transcripts detected in skeletal muscle tissue from melanized focal changes. (D) Phase contrast image for structure analysis in the region of interest (ROI). (E+F) Channel-2 (red) and 3 (purple) were unstained and no signal detected. Scale bar = 50µm


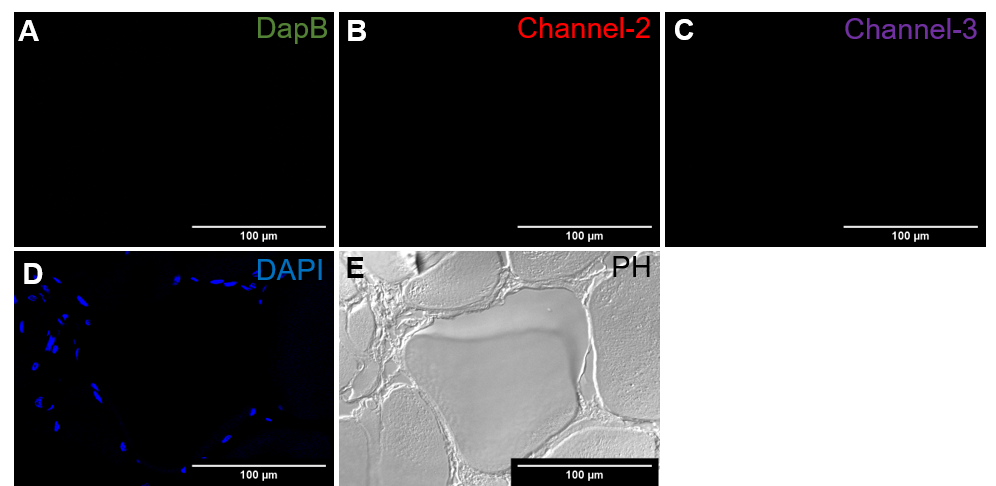


**Figure S2**. Negative Control for Fluorescent in situ hybridization (FISH) **(A)** No detection of DapB (green) positive staining. **(B+C)** Channel-2 (red) and channel-3 (purple) were unstained for any target. **(D)** Cellular nuclei DNA staining with DAPI (blue). **(E)** Phase contrast image for the myocyte structures. Scale bar = 100µm


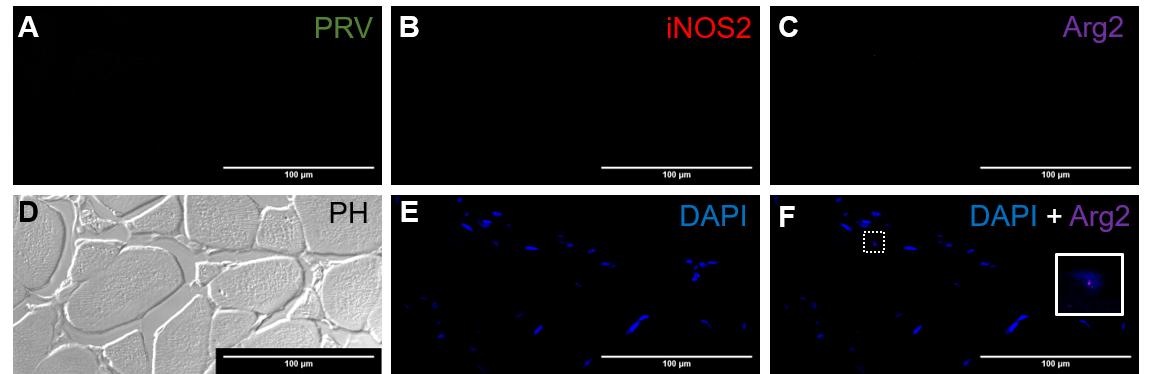
**Figure S3**. Red focal changes in uninfected fish **(A)** No PRV-1 (green) specific staining detected. **(B)** No iNOS2 (red) positive cells (M1) were stained. **(C+E+F)** negligible number of Arg2 (purple) positive cells (M2) were detected (inset). Nuclei DNA stained with DAPI (blue). **(D)** Phase contrast image showing myocytes and cells in between myocytes. Scale Bar = 100µm


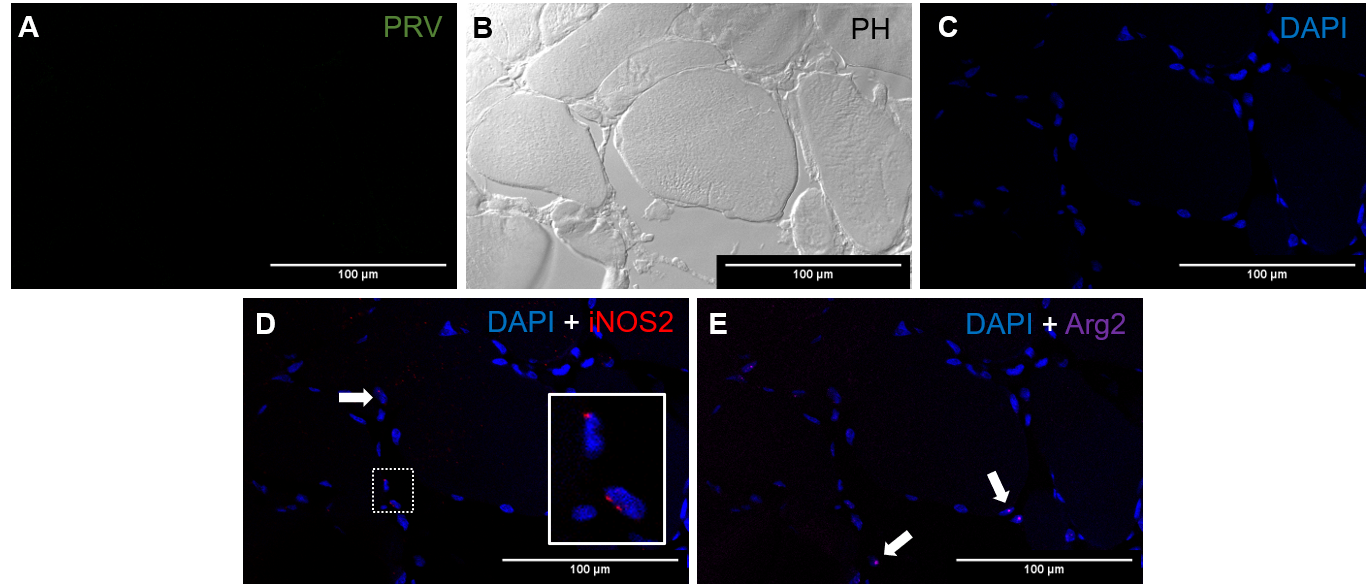


**Figure S4**. Melanized focal changes in uninfected fish **(A)** No PRV-1 (green) specific staining detected. **(B)** Negligible iNOS2 (red) positive cells (M1) were stained. **(C)** phase contrast image. **(D+E+F)** Very few of Arg2 (purple) positive cells (M2) were detected. Nuclei DNA stained with DAPI (blue). Scale Bar = 100µm


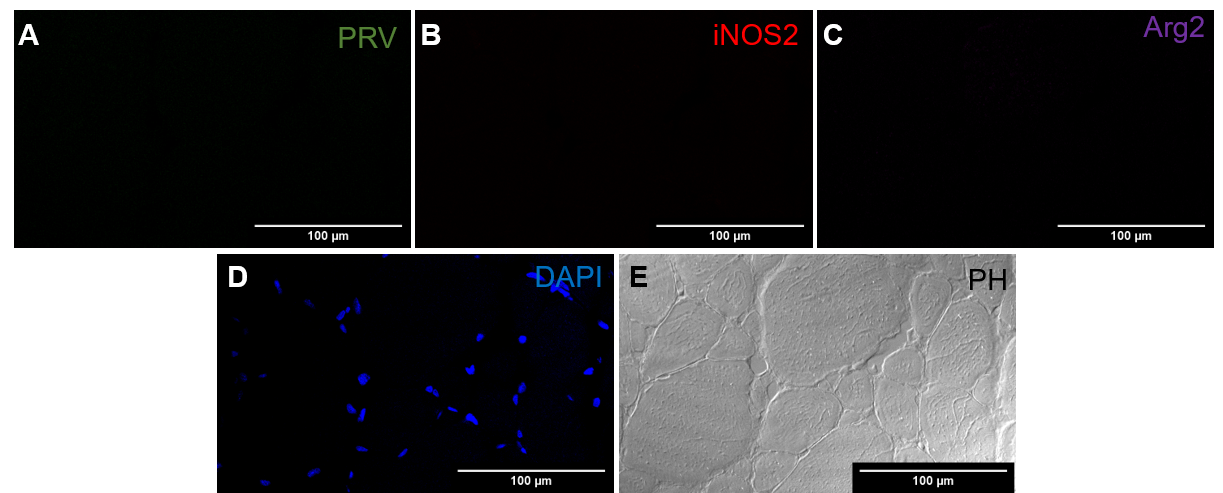


**Figure S5**. No focal changes in uninfected fish **(A)** No PRV-1 (green) specific staining detected. **(B)** No iNOS2 (red) positive cells (M1) were stained. **(C)** No Arg2 (purple) positive cells (M2) were detected. **(D)** Nuclei DNA stained with DAPI (blue). **(E)** Phase contrast image. Scale Bar = 100µm


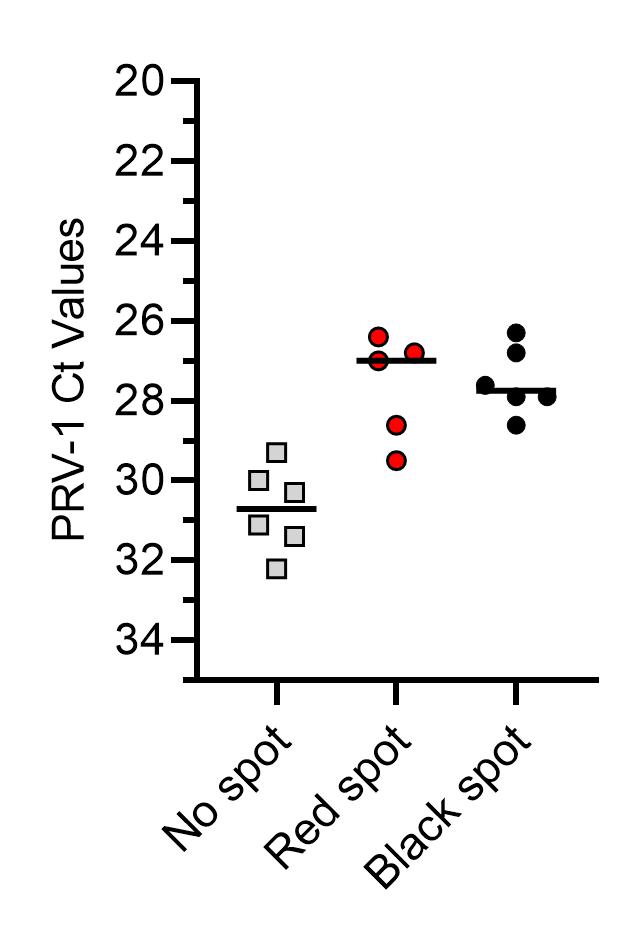


**Figure S6.** Ct values of PRV-1 in different spots categories detected by RT-qPCR. The lines show the median Ct value.

| **Sr. No.** | **Category** | **Individual fish** | **PRV-1 (Ct value)** | **Spots grading (1-3)** |
| --- | --- | --- | --- | --- |
|  | No Spot (uninfected) | Fish 1 | No Ct | - |
|  |  | Fish 2 |  |  |
|  |  | Fish 3 |  |  |
|  |  | Fish 4 |  |  |
|  | No Spot (infected) | Fish 1 | 29.3 | - |
|  |  | Fish 2 | 31.4 |  |
|  |  | Fish 3 | 30 |  |
|  |  | Fish 4 | 31.1 |  |
|  |  | Fish 3 | 32.2 |  |
|  |  | Fish 4 | 30.3 |  |
|  | Red spot (uninfected) | Fish 1 | No Ct | 2 |
|  |  | Fish 2 |  | 1 |
|  |  | Fish 3 |  | 3 |
|  |  | Fish 4 |  | 3 |
|  |  | Fish 3 |  | 2 |
|  |  | Fish 4 |  | 2 |
|  | Red spot (infected) | Fish 1 | 26.8 | 3 |
|  |  | Fish 2 | 29.5 | 2 |
|  |  | Fish 3 | 26.4 | 1 |
|  |  | Fish 4 | 27 | 3 |
|  |  | Fish 5 | 28.6 | 2 |
|  | Black spot (uninfected) | Fish 1 | No Ct | 1 |
|  |  | Fish 2 |  | 2 |
|  |  | Fish 3 |  | 2 |
|  |  | Fish 4 |  | 2 |
|  |  | Fish 3 |  | 2 |
|  |  | Fish 4 |  | 2 |
|  | Black spot (infected) | Fish 1 | 27.9 | 3 |
|  |  | Fish 2 | 27.6 | 3 |
|  |  | Fish 3 | 27.9 | 2 |
|  |  | Fish 4 | 26.3 | 1 |
|  |  | Fish 3 | 26.8 | 1 |
|  |  | Fish 4 | 28.6 | 2 |

Note: All skeletal muscle tissue samples were run for RT-qPCR gene expression analysis from each group.

**Table S1**. Details of the individual fish from all groups in relation to PRV-1 infection and grading of macroscopic lesions.
